# Gallstone ileus from a non-calcified stone: a challenging diagnosis

**DOI:** 10.1259/bjrcr.20170038

**Published:** 2017-05-10

**Authors:** Andrew Ian Goldfinch, Simon John Prowse

**Affiliations:** Department of Radiology, Lyell McEwin Hospital, Elizabeth Vale, Australia

## Abstract

Gallstone ileus is an uncommon and often life-threatening complication of cholelithiasis. In this case, we discuss a difficult diagnostic case of gallstone ileus with a non-calcified gallstone. An 88-year-old female presented with abdominal pain and vomiting. A CT scan was arranged and showed an evolving bowel obstruction although no frank hyperdensity suggestive of a gallstone was noted. Initially the cause of the bowel obstruction was uncertain, but after discussion with the treating team and further review of the images, the patient was diagnosed with gallstone ileus. The patient underwent emergency surgery and a 41 mm obstructing calculus was removed from the patient’s jejunum, later confirmed on histological diagnosis.

## Introduction

Gallstone ileus is a rare complication of choleliathiasis. It has a propensity to affect females and the elderly, and accounts for under 0.5% of cases of mechanical small bowel obstruction.^1,2^ It occurs as a gallstone enters the small bowel through a biliary enteric fistula, with more than half of these being cholecystoduodenal fistulas. This fistula is created as the gallstone exerts pressure against the biliary wall, leading to necrosis.^3^ A bowel obstruction then occurs if the newly entered gallstone is of sufficient size, usually greater than 2 cm.^4^ The ileum is the narrowest part of the bowel and is thus the area affected in the majority of cases.^2,5^

Clinically, gallstone ileus presents as an episodic bowel obstruction with diffuse abdominal pain and vomiting. Interval improvement in symptoms may occur as the stone becomes dislodged, with symptoms recurring as the stone becomes repeatedly obstructed.^5^ CT imaging is most often used in the investigation of gallstone ileus,^6–8^ as only a minority of gallstones have sufficient calcium content to be visible on abdominal X-rays.^9,10^ Management of gallstone ileus is predominantly surgical, usually involving an enterolithotomy with or without additional procedures.

## Case report

An 88-year-old female presented to the emergency department with abdominal pain and vomiting. She was mildly tender in her right upper quadrant but was focally more tender in her right lower quadrant. Her blood results showed an elevated white cell count of 12.9 ×  10^9 ^l^–1^ and a neutrophil count of 10.4 × 10^9^ l^–1^. Furthermore her C-reactive protein level was 34 mg/L. An abdominal ultrasound showed multiple calculi within the gallbladder. Gallbladder wall thickness was not assessed. A non-contrast CT scan, due to the patients impaired renal function, was then ordered as the patient’s physical exam findings were not in keeping with a diagnosis of cholecystitis.

**Figure 1.**
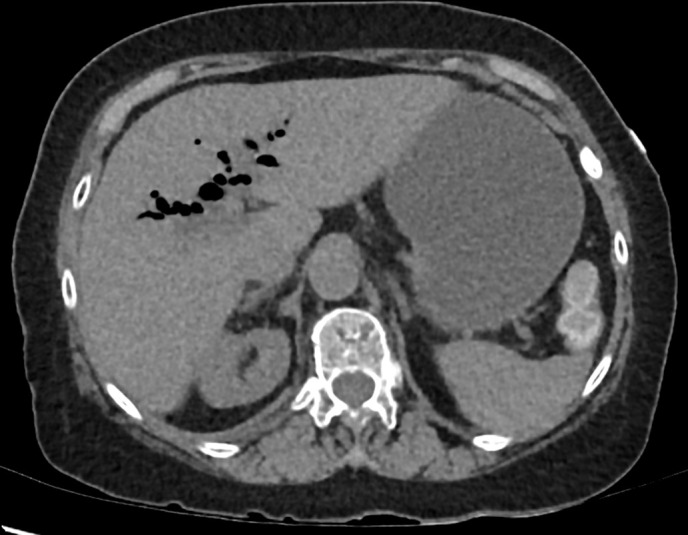
Non-contrast CT abdomen and pelvis. Air is demonstrated in the biliary tree. This was initially attributed to post sphincterotomy pneumobilia.

The CT showed abnormal dilatation of the proximal small bowel with a possible transition point in the left iliac fossa. Pneumobilia was also noted due to prominence of the hepatic biliary tree (Figure 1). No evidence of a ductal calculus was observed. The CT scan was reported as showing features of a bowel obstruction with a transition point in the left iliac fossa. The pneumobilia was reported as being possibly an indicator of previous sphincterotomy. There was no evidence of appendicitis or another inflammatory process in the right iliac fossa.

**Figure 2.**
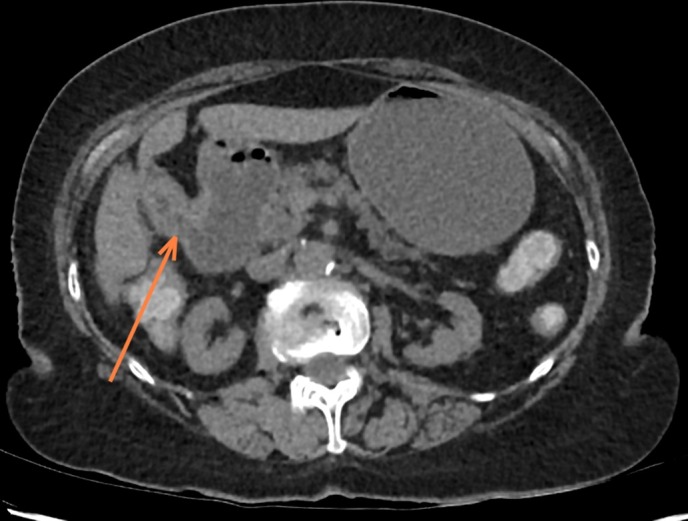
The arrow marks the location of the choledochoduodenal fistula. A continuous low density tract can be appreciated between the contracted gallbladder and the adjacent duodenum.

**Figure 3.**
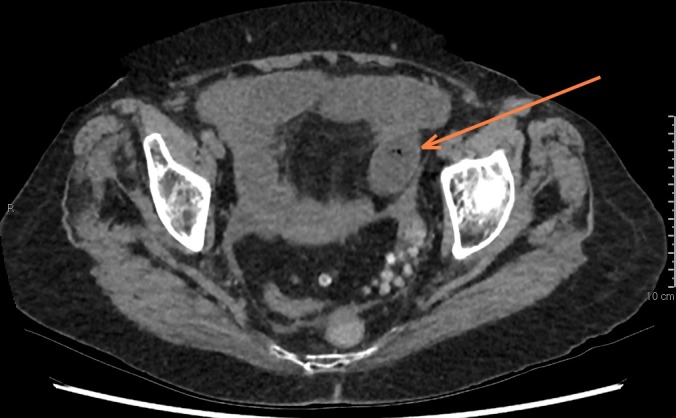
The arrow is marking the transition point in the left iliac fossa and what is likely an obstructing non-calcified gallstone. Gas can be appreciated within the gallstone. Following surgical removal, this gallstone measured 41 × 25 mm.

The following day, while proceeding with conservative management, the treating team requested a small bowel follow through to investigate the obstruction. After further review and discussion with the treating team it was revealed that there was no history of an endoscopic retrograde cholangio-pancreatography or cholecystectomy. An addendum was added to the CT report, suggesting that the appearances were indicative of a gallstone ileus from a choledochoduodenal fistula (Figure 2) with a non-calcified gallstone possibly being present at the transition point in the left iliac fossa (Figure 3).

The patient underwent a laparotomy and a gallstone was found obstructing the distal jejunum. A 41 mm gallstone was removed by vertical enterotomy, later confirmed on histological diagnosis. The patient had an uneventful recovery in hospital and was discharged a few days later to a regional hospital for rehabilitation. A cholecystectomy or fistula closure was not performed and she was not referred for further surgical follow-up on discharge, likely due to the patient’s advanced age and medical comorbidities.

## Discussion

This case represents what was initially a missed diagnosis of gallstone ileus due to a non-calcified gallstone. The pneumobilia was at first reported as being due to a previous sphincterotomy. The diagnosis of gallstone ileus was made later on consultation with the treating team.

The CT findings of gallstone ileus include thickening of the gallbladder wall, pneumobilia and bowel obstruction with obstructing gallstones.^6–8^ However, gallstones may be missed on CT as they are not always hyperdense.^9,10^ In one study, low density stones accounted for around 3% of gallstones.^9^ In this case, gas was appreciated within the centre of the non-calcified gallstone. This is a well-documented sign of gallstones in the literature.^11 ^ In addition to this, a cholecystoduodenal fistula could be seen on CT.

Management of gallstone ileus involves removal of the obstructing stone leading to resolution of the bowel obstruction. This is achieved through either a laparoscopic or open enterolithotomy. It is not uncommon for a bowel resection to be required if surgical complications such as perforation or difficult stone retrieval occur.^5^ A definitive procedure to treat the cause of the ileus, such as a cholecystectomy or fistula closure is also often required, to reduce other complications such as recurrence or cholecystitis.^5^ This procedure can often be performed at the same time as an enterolithotomy in low-risk patients.^2,12,13^ Higher risk patients, such as in the case discussed, may undergo enterolithotomy without an additional procedure as fistulas may close or reduce in size without subsequent intervention.^14,15^

Gallstone ileus remains an important diagnosis as it results in significant morbidity and mortality. The mortality rate remains high at around 5–7%.^1,14,16^ Furthermore, the mortality rate is 5 to 10 times higher than other mechanical causes of small bowel obstruction.^1^ Recurrent gallstone ileus occurs in a significant minority of patients treated with enterolithotomy alone, and more than half of these occur in the first 6 months following surgery.^14^

This case emphasizes the importance in considering gallstone ileus as a cause for a mechanical bowel obstruction. It demonstrates a difficult diagnostic case of gallstone ileus with no evidence of calcification or increased density on CT that would be suggestive of an obstructing gallstone. The radiological diagnosis of gallstone ileus certainly changed management immediately, and may have saved this frail patient. Difficulty in diagnosis was further compounded by conflicting physical examination findings with a seemingly inconclusive abdominal ultrasound study. There is a need for careful consideration of clinical history in conjunction with other radiological signs such as pneumobilia or bowel obstruction to establish a diagnosis of gallstone ileus.

## Learning points

1. Gallstone ileus is a serious cause of mechanical bowel obstruction, often presenting as episodic abdominal pain and vomiting.
2. In gallstone ileus, gallstones enter the small bowel through a biliary enteric fistula.
3. A CT scan is chosen imaging method to diagnose gallstone ileus, but not all gallstones are immediately evident on imaging.
4. The CT findings of gallstone ileus can include thickening of the gallbladder wall, pneumobilia and bowel obstruction with obstructing gallstones.
5. The management of gallstone ileus is predominantly surgical, with enterolithotomy usually followed with a procedure such as cholecystectomy or fistula closure.

## Acknowledgements

The authors certify that they have no affiliation with any organization with any financial or non-financial interest in the subject matter discussed.

## Consent

Informed consent for the case to be published (including images, case history and data) was obtained from the patient(s) for publication of this case report, including accompanying images.
